# On the Rapidity and Extent of the Physical and Chemical Changes in the Interior of the Body

**Published:** 1859-09

**Authors:** John C. Dalton

**Affiliations:** Professor of Physiology and Microscopic Anatomy in the College of Physicians and Surgeons of New York


### ECLECTIC DEPARTMENT,

### AND SPIRIT OF THE MEDICAL PERIODICAL PRESS.

*On the Rapidity and Extent of the Physical and Chemical Changes in the Interior of the Body.* By JOHN C. DALTON, JR., M. D., Professor of Physiology and Microscopic Anatomy in the College of Physicians and Surgeons of New York. Read before the New York Academy of Medicine, March 16, 1859.

OF all the departments of Physiology, there are none which have made greater progress of late years than that which relates to the accurate investigation of the physical and chemical phenomena of the living organism. There is, also, no department of the science, which has a more direct and important bearing upon practical medicine. The study of these phenomena, like that of most others in which the physician is interested, has been obliged to pass through a certain period of infancy and progressive development. It required a very considerable time, and no little expenditure of ill-directed and apparently fruitless investigation, before physiologists came to understand the proper methods even of extracting and examining the substances which constitute the animal frame. The true mode of investigation having been finally ascertained, and the way fairly opened for profitable study in this direction, a long series of labors followed, which yielded the most valuable results, and which have now made us tolerably well acquainted with all the more important proximate principles to be found in the interior of the frame, the substances taken with the food and in respiration, and the excrementitious matters discharged from the body.

A similar difficulty and delay were experienced, also, in the early study of the physiological relations of these different materials, and their mutual reaction in the vital processes. Numerous as they are, and of widely different nature and properties, and mingled together in varying proportions in the animal tissues and fluids, the phenomena which they presented were of a novel and complicated character, and could not, at first, be readily appreciated. The usual methods of examination and experiment, which had been habitually employed by chemists in the study of isolated inorganic substances, failed of accomplishing their purpose when applied to this new order of phenomena. These methods, therefore, led only to imperfect or contradictory results. The insufficiency of the means first used being at last made manifest, it was by a very gradual process that other and more appropriate methods were substituted for them.

Such a change, however, was absolutely necessary before any completely satisfactory progress could be made; and, singular as it may seem, it is comparatively within a recent period that we have come to a full understanding of the direction in which this kind of physiological investigation should be pursued. The study of the vital phenomena, in fact, can be profitably followed only by the direct examination of these phenomena, *as they actually take place in the living body.* This is the only field in which physiological questions can be positively decided, and in which the labors of the experimenter can be made actually serviceable to the progress of medicine. For, however much assistance we may derive from comparative experiment and scientific analogies, the results obtained from these sources can only be of service by suggesting probabilities, and as preparatory to the final investigation. The actual phenomena presented in the living body often turn out, in fact, to be quite different from any which could have been previously anticipated.

Whenever a wider field is thus opened to the laborers in any department of natural science, and more efficient methods of investigation brought into use, the subject is often so much modified as to present itself under an entirely new aspect, and becomes gradually extended by the development of those portions which were previously incomplete. While this process is going on, the new order of facts, and the new ideas which originate from them, remain, for a while, partially concealed among the numerous isolated discoveries which have made their appearance from time to time. During this period, therefore, they do not exist as connected statements, in the works of any writer, but are only to be found scattered here and there, exerting an important influence on the immediate progress of the science, but not yet fully recognized as forming a part of its acknowledged doctrines.

Something of this kind is now taking place in the department of physiology to which I have alluded. After becoming acquainted with the nature and properties of the proximate principles, or ingredients, of the body, and learning the true method of investigating their changes and reactions, it soon became evident that the mutual dependence and physiological relations of these materials could not be fully understood without knowing something about their absolute and relative *quantities.* The methods of investigation already coming into general use enabled experimenters to overcome, in a great measure, the obstacles presented by this new and difficult portion of the subject; and the success of these labors, so far as they have yet been pursued, promises results hardly inferior to those which have been derived from the accurate quantitative analysis of the inorganic chemists.

I have accordingly thought that the duty which has been assigned to me for this evening, by the Council of the Academy, could not be better discharged than by asking your attention, as the subject for our present consideration, to the *Rapidity and Extent of the Physical and Chemical Changes in the Interior of the Body.*

I would remark, in the outset, that both the quantity of the animal fluids, and the rapidity of the changes which they undergo, are found, upon direct investigation, to be very much greater than we should have anticipated. We have known, for a long time, that the animal body is the seat of incessant absorption and exhalation, decomposition and metamorphosis, and that these phenomena are characteristic of living beings, necessary as the conditions of their existence, and directly dependent on their structure and organization; but the real extent of these changes, the actual amount of the substances absorbed and discharged, produced and destroyed, in the body, when measured by experiment, far surpasses our previous expectation, and gives us a new and much larger comprehension of the true nature of the vital operations.

Quantitative experiments of this kind are most readily made upon the external operations of supply and waste, or those of nutrition and excretion, performed by the body as a whole. The air we breathe, the food and water taken into the alimentary canal, together with the fluids and solids discharged with the urine, feces, and perspiration, and the gaseous exhalations of the breath,—these form the two compensating quantities of ingesta and egesta, which when properly balanced, maintain the entire organism in a healthy condition.

The process of *Respiration*, being the most incessant and uniform of those just mentioned, requires to be examined first.

The history of experiment on this subject would illustrate in a very forcible manner the obstacles to be encountered in nearly all quantitative investigations of the animal functions. For mechanical difficulties present themselves in the way of such experiments, both more numerous and more troublesome than would be supposed, even in so apparently simple a matter as that of measuring accurately the different gases absorbed and exhaled with the breath. I will leave out of the question, however, the explanation of these difficulties, and of the manner in which they have been overcome, and pass at once to a brief statement of the results which have been finally obtained.

An adult man, of average size and development, takes into the lungs with each ordinary inspiration, twenty cubic inches of atmospheric air. Since he breathes usually eighteen times a minute, this makes 360 cubic inches of air inspired per minute, 21,600 cubic inches per hour, and 518,400 cubic inches per day. As the respiration is increased, however, in rapidity and intensity, with every muscular exertion, the actual daily quantity of air used in respiration is not less than 600,000 cubic inches, or 350 cubic feet. I give these quantities in round numbers, since these are more easily understood, and since the physiological variations in respiration are such that an absolutely precise estimate would be both unnecessary and inapplicable; 350 cubic feet, therefore, represents the quantity of atmospheric air daily used in respiration. During this process the air loses, upon the average, 5 per cent. of its volume of oxygen. The total quantity of oxygen, therefore, actually absorbed by an adult man, during twenty-four hours, is seventeen and a half cubic feet, or about four times the bulk of his own body.*

The quantity of carbonic acid exhaled is but little less than this. The expired air usually contains about four per cent. of its volume of carbonic acid. According to the researches of Vierordt, and Andral and Gavarret, the average quantity of this gas exhaled is very nearly 1,150 cubic inches per hour, or fifteen and a half cubic feet per day.† The volume of car-

---

* This equals, in weight, 7,134 grains, or a little over one pound.

† This is, by weight, 10,740 grains, or a little over one pound and a half.

bonic acid exhaled with the breath, is necessarily somewhat less than that of the oxygen absorbed, since a certain portion of oxygen is finally disposed of in the body in the formation of other excretory substances, and accordingly does not reappear under the form of carbonic acid. The proportion of surplus oxygen thus employed varies somewhat in different animals, and with the use of different kinds of food; but the above numbers represent the quantities of the two substances in the human species, under ordinary conditions and regimen.

In some instances the intensity of respiration is considerably greater than this; and particularly in small animals, endowed with well-marked nervous excitability. Regnault and Reiset, found that the consumption of oxygen, for a given time, in proportion to the whole mass of the body, was more abundant in small animals and in birds, than in the human subject; and that in sparrows and greenfinches, it was from eight to ten times greater than in fowls and ducks.

The entire quantity of *food and drink* consumed in twenty-four hours may be ascertained, without much difficulty, by confining the diet to a few simple and nutritious articles. For a healthy and vigorous man, taking free exercise in the open air, the average amount of food required per day, is as follows:—

| | | | | |
|---|---|---|---|---|
| Meat | 16 ounces or | 1.00 | lb. | avoirdupois. |
| Bread | 19 " | " 1.19 | " | " |
| Butter or Fat | 3½ " | " 0.22 | " | " |
| Water | 52 fluid oz. " | 3.38 | " | " |

Beside the fluids taken as drink, however, the meat and vegetable substances, used as food, themselves contain a very considerable proportion of water, which may be determined by analysis. By uniting these two estimates, the total quantity of water absorbed daily, is increased to a little over four pounds and a quarter; while the various solid ingredient of the food may be ascertained separately, by a similar process.

A corresponding quantity of material is discharged daily, by the excretions and exhalations. A little over two pounds of water passes off with the urine. Lavoisier and Seguin found that nearly as much is discharged daily with the perspiration; and a certain amount is also exhaled, under the form of aqueous vapor, with the breath. It has been found difficult to measure exactly this last quantity, but it appears, from the most reliable data which we possess, that the watery vapor exhaled from the lungs, is at least one-fifth as abundant as that discharged from the skin.

If we now bring into the calculation, the quantities of urea and saline substances discharged with the urine, and the insoluble matters of the intestinal secretions, all easily ascertained by experiment, we find that the total amount of ingesta and egesta, for twenty-four hours, is that expressed in the following table:—

| ABSORBED DURING TWENTY-FOUR HOURS. | | DISCHARGED DURING TWENTY-FOUR HOURS. | |
|---|---|---|---|
| Oxygen | 1.019 lbs. | Carbonic acid | 1.535 lbs. |
| Water | 4.275 " | Aqueous vapor | 0.445 " |
| Albuminous Matter | .340 " | Perspiration | 1.965 " |
| Starch | .590 " | Water of the Urine | 2.020 " |
| Fat | .220 " | Urea and Salts | .150 " |
| Salts | .056 " | Feces | .385 " |
| | 6.500 " | | 6.500 " |

No less than six and a half pounds, therefore, are absorbed and discharged daily by the healthy adult human subject; and for a man having the average weight of 140 pounds, in twenty-two days a quantity of material thus passes through the system, equal to the weight of the entire body.

It must be remarked, also, that this is not a simple phenomenon of the passage, or filtration, of foreign substances through the animal frame. The materials which are absorbed actually combine with the tissues, and form a part of their substance; and it is only after undergoing subsequent decomposition, that they finally make their appearance in the excretions. None of the solid ingredients of the food are discharged under their own form in the urine, viz. as starch, fat, or albumen; but are replaced by urea and other crystallizable substances, of a different nature. Even the carbonic acid exhaled by the breath, as experience has now taught us, is not produced by a direct oxidation of carbon; but originates by a steady process of decomposition, throughout the tissues of the body, somewhat similar to that by which it is generated in the decomposition of sugar by fermentation. These phenomena, therefore, indicate an actual renovation in the substance of which the body is composed.

The quantity of material used in this manner for the purposes of nutrition is, in some instances, much larger than the above. A vigorous dog, weighing thirty pounds, will readily consume in a single day two pounds of fresh meat, a quantity of solid food nearly four times greater, in proportion to the size of the body, than that used by man; and young birds have been known to consume daily as much as one half, or even three quarters, of their entire weight.

Such is the activity of the most palpable changes to which the body is subject, viz. the external phenomena of supply and waste. But between these two extremes there exists a series of internal changes, incessantly going on in the body, and constituting the processes of circulation, secretion, and nutrition. Investigation shows that the activity of these internal phenomena is much greater than that of the others just described.

The difficulties in the way of ascertaining the exact quantity of *blood* in the body, depends in great measure upon the rapidity of these internal changes. It is impossible to ascertain this quantity by merely opening the large arteries and veins of an animal, and collecting all the blood which escapes; for the body can never be completely drained of blood by this means, since some of it always remains behind in the blood-vessels of the internal organs. Valentin and some others have, accordingly, endeavored to settle the question by drawing a certain quantity of blood from the veins, then injecting a similar amount of distilled water or saline solution of known strength, afterward performing a second venesection, and finally determining the difference in density between the first and second specimens of blood.

This plan might succeed if the blood-vessels were a closed circuit of impermeable tubes, through which the blood moved without alteration or modification in the different organs. But this is not the case. The blood suffers constant changes, both in density and composition, in different parts of the circulation. The mere withdrawal of a certain portion of it from the veins causes an immediate alteration in the density of the remainder.

As the vessels are partially drained by venesection, they are immediately filled up again, to a certain extent, by the imbibition of serous fluid from the neighboring tissues. Becquerel and Rodier found that this alteration of the blood was very well marked, in the human subject, even within the limits of a single moderate bleeding. It is a continuous and progressive alteration, "beginning," as they express it, "with the first drop which escapes, and ending only with the last." These observers found that in bleeding from the arm, to the extent only of twelve and a half ounces, the portions of blood drawn toward the end of the operation were already different in density from those which escaped at the beginning; the proportion of solid matters having fallen, in some instances, from 167 to 163 parts per thousand, and that of the fibrin from 2.42 to 2.17 per thousand.

All such methods, therefore, of determining the quantity of blood are necessarily uncertain. The best plan yet suggested is that employed by Lehmann and Weber. They collected all the blood which escaped from the vessels of two criminals, executed by decapitation. They then injected the arteries of the head and trunk with distilled water, and collected the bloody fluid which returned by the veins; and, by ascertaining the quantity of solid matter which this fluid held in solution, they calculated the quantity of blood which had remained in the vessels of the internal organs. This amount, added to the former, gave the entire quantity of blood in the body.

This plan, even, is not altogether free from objection; since, when the vessels of the body are injected after death, the water not only transudes and infiltrates the neighboring parts, but it also absorbs various substances from the solid tissues, in proportions different from those in which they would have been taken up by the blood. Still, it is sufficiently accurate for ordinary purposes; and the result thus obtained is, that for a man of average size, the entire quantity of blood in the body is from seventeen to eighteen pounds.

But this blood is not stationary, but in motion. The rapidity of its circulation, as ascertained by experiment, forms one of the most remarkable phenomena of the living body; and its discovery, when first made, naturally excited the attention of the profession by its very singular and unexpected results. These results are well known, and only require to be mentioned here in a brief manner. Volkmann, Poisseuille, Hering, and Blake injected into the jugular vein of the horse certain saline substances, easily recognizable by chemical tests, such as ferrocyanide of potassium and nitrate of baryta, and then examined the blood drawn from the jugular of the opposite side at intervals of five seconds afterward; detecting the injected substance by this means after it had passed downward to the heart, through the pulmonary capillaries, upward by the carotid arteries, and again downward by the vein. They thus found that it traversed the entire round of the circulation in from twenty to twenty-five seconds.

It was at first thought that this statement could not be the expression of the truth, and that some source of deception must have existed in the details of the experiment; but subsequent investigation failed to discover any other mode of accounting for the appearances observed; and it was finally conceded that the substance injected must have passed with the blood through the circulation, in that short period of time.

These experiments have the great advantage of having been performed without opening any of the great cavities of the body, or exposing the circulatory organs to the unnatural contact of the air. Even here, however, it is not possible to fix upon the exact period required by the blood for its complete passage through the circulation, but only to make an approximative calculation. For it was found that certain other substances, injected at the same time, such as nitrate of potass, acetate of ammonia, or a little alcohol, hastened or retarded to a certain extent the appearance of the ferrocyanide in the opposite jugular. It is plain, therefore, that the rapidity of the circulation does not depend simply on the impulse of the arterial current, but is influenced also by the relations existing between the physical constitution of the blood and that of the walls of the capillaries and the neighboring tissues. This will become still more evident when we have examined some of the other conditions and phenomena belonging to the circulation. The above variations, however, in all the experiments performed, were confined within the limits of from eighteen to forty-five seconds.

Another question of somewhat similar nature, relates to the quantity of blood passing through the heart and principal vessels in a given time. To determine this point, we must first know the quantity of blood expelled from the left ventricle with each cardiac pulsation. This cannot be ascertained by simply taking the capacity of the ventricle, after death, by artificial injection. For, in the first place, such an artificial injection will distend the ventricle to a much greater degree than it is ever filled by the blood during the normal condition of the circulation; and secondly, it is not at all certain, as assumed by some writers, that the ventricle empties itself completely at each pulsation. It is my own impression, from watching the pulsations of the heart, exposed in the chest of the living animal, that its emptying is partial only, and that, like the arteries, it always contains a certain amount of blood, when contracted as well as in a state of dilatation.

The only direct mode of determining the question which now occupies us, is to divide suddenly the arch of the aorta by a transverse section, and to measure the quantity of blood discharged during the first few seconds afterward. The objection to this plan is, that the opening of the chest and exposure of the thoracic organs diminishes, very probably, the force of the heart's action, and lessens, accordingly, the quantity of blood expelled at each pulsation. The result thus obtained, however, in this case, will certainly not be exaggerated, but, if anything, will fall below the truth.

I have exposed the heart, in this manner, in a dog weighing fourteen and a half pounds, after producing insensibility by the inoculation of woorara, and keeping up artificial respiration by means of a bellows inserted into the trachea. I find that in cutting across the aorta, just above its origin from the left ventricle, 300 grains of blood escape from the divided orifice in fifteen seconds. This will give 1,200 grains per minute, 72,000 grains per hour, and 1,728,000 grains per day. In an animal of that size, therefore, the quantity of blood passing through the heart in twenty-four hours is at least 247 pounds, or about seventeen times the weight of the whole body. Applying this result to an adult man, weighing 140 pounds, we find that the quantity of blood passing daily through the heart is at least 2,380 pounds.

It is this remarkable activity of the circulation which accounts for the large quantity of the internal secretions, constantly produced and reabsorbed in various parts of the body. The saliva is one of the secretions which are produced in considerable abundance. The only difficulty in ascertaining its exact quantity lies in the varying activity of its production at different times. Under ordinary conditions the saliva is merely secreted in sufficient amount to keep the mucous surfaces of the mouth moist and pliable, and to preserve the fauces and pharynx in a fit state to assist in the functions of the voice and respiration. But any gentle irritation, and particularly the introduction and mastication of food, will at once increase it to four or five times its usual amount. As this extra quantity, however, is naturally swallowed with the food, it cannot be directly obtained and measured. I have found that the saliva may be collected from the mouth, without using any artificial stimulus, to the amount of 556 grains per hour. By weighing a small quantity of bread and meat, both before and after mastication, it appears that bread gains during this process fifty-five per cent. of its weight by the absorption of saliva; and that freshly-cooked meat gains, from the same cause, forty-eight per cent. of its weight. We have already seen that the daily allowance of these two substances for a man in full health, is nineteen ounces of bread and sixteen ounces of meat. The entire quantity of saliva required for mastication is therefore 8,932 grains. The quantity secreted in the intervals between meals, for twenty-two hours, at 556 grains per hour, is 12,232 grains; and the entire daily quantity of the secretion, thus ascertained, is 21,164 grains, or a trifle over three pounds avoirdupois.

The gastric juice is much more abundant. Like the saliva, it is secreted, during the intervals of digestion, in very small quantity; hardly sufficient, in some instances, to preserve the normal acid reaction of the gastric mucous membrane. It is immediately poured out, however, in great abundance on the introduction of food, and continues to be secreted during the entire process of digestion. In the dog, I have found it to be produced with such rapidity, shortly after feeding, that from 2 to 2½ fluid ounces could be collected, from a gastric fistula, in the course of 15 minutes. Bidder and Schmidt actually collected, from a dog weighing 34 pounds, in a total period of 12 hours, one pound and three quarters of gastric juice; and by applying the same calculation to a man of medium size, the authors estimate the total daily quantity in the human subject, as a little less than 14 pounds.

An approximation to this result may also be obtained in a little different manner, viz., by ascertaining the quantity of gastric juice necessary to digest all the albuminoid matters taken with the food. Such an experiment, on the artificial digestion of a known weight of muscular flesh by a given quantity of gastric juice, shows that about 33 grains of meat are dissolved by each fluid ounce of gastric juice. One pound of meat, the daily quantity used by a man in good health will therefore require 13¼ pints of gastric juice; and as a certain amount of albuminoid matter exists also in the bread and other vegetable substances used as food, there is every reason to believe that Bidder and Schmidt's estimate, of 14 pounds per day, is certainly not above the quantity actually produced.

By similar experiments, the daily quantity of the bile and of pancreatic juice has been ascertained with close approximation to certainty. Bidder and Schmidt find that for every pound weight of the whole body, there is secreted in the cat 102 grains of bile, in the dog 140 grains, in the sheep 178, and in the rabbit 958 grains per day. This secretion is therefore more abundant in vegetable feeders than in the carnivorous animals; and, taking the lower standard as a basis for calculating the amount of bile in the human subject, we find that the total daily quantity is about 17,000 grains, or very nearly 2½ pounds.

The quantity of pancreatic juice, so far as ascertained, appears to be considerably less than the foregoing. I have not been able to obtain from the larger of the two pancreatic ducts, in the dog, more than 5 or 6 fluid drachms during a period of several hours. Bidder and Schmidt collected from the same animal, on the average 14½ grains per hour; from which they calculate the daily quantity in man as about 2,500 grains, or a little over one third of a pound.

The intestinal juice, or the product of the follicles of Lieberkuhn, has never been estimated as to quantity, since the mechanical difficulties in the way of obtaining any experimental data with regard to it are such, that no contrivance has yet been able to overcome them. There is every probability, however, that its quantity is tolerably large, since it is secreted from the walls of a tubular canal 20 to 25 feet in length, and presenting an internal surface of at least 7 to 8 square feet, studded throughout with secreting follicles.

The entire quantities of the above secretions, so far as they have yet been examined, amount, therefore, to between 19 and 20 pounds per day. This large quantity of the secreted fluids has sometimes been regarded as incredible, because the exact nature of the process has been misconceived. These fluids are not drained away from the body, and their materials lost to the circulation, but they are all reabsorbed, after having been exuded into the cavity of the alimentary canal. They are all, in some sense, digestive fluids, whose function is, for the most part, to act upon and dissolve the food; and as soon as this is accomplished, they are again taken up by absorption, together with the products of digestion which they have dissolved. Their secretion and reabsorption, in fact, go on continuously and simultaneously; successive portions passing and repassing through the glandular apparatus and the intestinal mucous membranes. There is no drainage, therefore, exerted upon the system by this process; but only a kind of internal renovation and interchange of the animal fluids, simultaneous with and secondary to the circulation of blood in the blood-vessels.

Let us now examine, for a moment, the nature and activity of the process by which this secretion and reabsorption take place. How is it that the animal fluids exude through the mucous membranes, and again penetrate their substance to re-enter the circulation?

The phenomena in question have long been known to us under the names of *endosmosis* and *exosmosis*; and yet their exact nature has not, I think, been always accurately described. When an animal membrane, sufficiently recent to retain its natural texture and constitution, is placed, as a diaphragm, between certain liquids occupying distinct cavities, the two liquids are found, after a time, to have mingled with each other, more or less freely, on the two sides of the membranous partition. There is usually a passage of one liquid toward the other in large quantity, known as *endosmosis*, and also a passage of the second liquid toward the first, in smaller quantity, known as *exosmosis*. Now, this is not merely an act of simple filtration through pores, nor a mere admixture and diffusion of the two liquids through each other. Experience has shown that the conditions regulating endosmosis and exosmosis do not depend, as was formerly supposed, upon the difference in density of the two liquids, nor even upon their affinity for each other, but upon their affinity for the substance of the membrane between them. It is not the two liquids which act upon each other, but the membrane itself which acts upon the two liquids; and the liquids, in passing through the membrane, unite intimately with its substance, and form, for the time, an integral part of its texture.

We can understand, then, why a living animal membrane, so far from being an obstacle to the passage of a liquid in any particular direction, may, on the contrary, favor this passage, and even be the active agent by which it is accomplished. All the phenomena, accordingly, of exudation and absorption in the living body, are instances of the exercise of this property, and depend upon it as the principal condition of their accomplishment.

This process is in reality much more active than we should be led to anticipate from our experiments with endosmometers in the laboratory. M. Gosselin, President of the Society of Surgery, at Paris, reported to the French Academy of Medicine, in August, 1855,* the following experiments on the rapidity of endosmosis in the tissues of the eye-ball. He dropped upon the cornea of the left eye of a rabbit, a watery solution of iodide of potassium, ninety grains to the ounce. At the end of seven minutes from the commencement of the experiment, he extracted both eyeballs from the animal, and examined, first the left and then the right eye in the following manner. The surfaces of the eyeballs were first washed with acidulated water, and the washings, tested with a solution of starch, presented no trace of the presence of iodine. None of the iodine, therefore, adhered to the external surface of the eyeball. The cornea was then detached, dried on both sides with a bit of linen, cut in pieces, bruised and macerated for a short time in a capsule with a little distilled water. This fluid, then tested by starch and nitric acid, showed a distinct blue coloration of iodide of starch. The crystalline, vitreous body, and iris of the same eye, examined in the same way, did not give so distinct a reaction. The opposite eye, subjected to similar tests, did not show the least trace of the presence of iodine.

In another experiment, the eye was extracted eleven minutes after the application of the solution of iodine to the conjunctiva. The cornea, aqueous humor, iris, sclerotic, crystalline, and vitreous body, in this case, all showed very evident indications of the presence of iodine; while in the opposite eye no such indications were found in any part.

In these instances, the solution of iodide of potassium had passed, by endosmosis, into the substance of the cornea in seven minutes, and in eleven minutes had penetrated through it into all the textures of the eyeball. In other experiments, the cornea and aqueous humor both contained

* Gazette Hebdomadaire, Sept. 7th, and 28th, 1855.

iodine in six minutes, four minutes, and three minutes after its external application; and in another still, the cornea alone presented faint but unmistakable indications of its presence at the end of a minute and a half.

In the above experiments, it is plain that the iodine actually passed into the deeper portions of the eyeball by imbibition and endosmosis, and was not transported from the conjunctiva by the vessels of the general circulation; since the tissues of the opposite eye, examined at the same time, showed no trace of its presence.

It is in this way that a solution of belladonna or of atropine, dropped upon the conjunctiva, penetrates the cornea, is taken up by the aqueous humor, and acts directly and locally upon the muscular fibres of the iris, without affecting the system at large, or producing any alteration in the eye of the opposite side. M. Gosselin applied a solution of sulphate of atropine to both eyes of two rabbits. In half an hour the pupils were dilated. Three quarters of an hour later, the aqueous humor was collected by puncturing the cornea with a trocar; and this aqueous humor, dropped into the left eye of a cat, produced, in half an hour, dilatation and immobility of the pupil confined to that side. These and other similar trials showed, beyond the possibility of doubt, that the aqueous humor of the affected eye actually contains atropine, while this substance is not present in the eye of the opposite side.

But these experiments, however ingeniously contrived, are clumsy and imperfect when compared with the anatomical conditions furnished by the organs of secretion and circulation in the living body. The complicated involution of the glandular surfaces, and the minute interlacement of follicles and blood-vessels, increase, to a remarkable degree, their extent of contact with each other, and consequently heighten the activity of their mutual reactions.

Each perspiratory gland of the skin, for example, consists of a minute coiled tube, which, when unravelled, is one-fifteenth of an inch in length. On the posterior portions of the body and limbs, there are about 500 of these glands to the square inch, on the anterior portions 1,000 to the square inch, and on the palm of the hand and the sole of the foot, 2,700 in the same space. The whole number of perspiratory glands in the body is at least 2,300,000, and the entire length of glandular tubing is accordingly not less than 153,000 inches, or about two miles and a half. It is easy to understand, therefore, that while each perspiratory gland secretes but a small quantity of fluid at a time, the entire amount produced by all of them may be very large.

The subdivision of the circulating currents in the capillary blood-vessels is equally minute. It is estimated that the united area of all the capillaries in the body is between three and four hundred times that of the arteries. It does not follow from this that there is three or four hundred times as much blood contained at once in the capillaries as in the entire arterial system; for although the united *area* of the capillary vessels is very large, their *length* is very small. The current of blood has only a short distance to pass over, from the termination of the arteries to the commencement of the veins; but, in making this passage, it is spread out into a multitude of minute streams. The effect of this anatomical arrangement is accordingly to disseminate a comparatively small quantity of blood over a very large space; and when we remember the readiness with which atropine and iodide of potassium penetrate the thick cornea into the interior of the eye, we can readily understand the instantaneous reaction which may take place between the blood, circulating in the capillaries of a glandular organ, and the substances contained in its tissues.

Bernard inserted a silver canula into the parotid duct on the two opposite sides of the face in the same dog. He then injected into the glandular tissue, through the canula on the left side, a solution of iodide of potassium. Immediately afterward, on exciting the secretion of the parotid gland on the right side, the first drops of saliva which flowed from the canula contained evident traces of iodine. In this period, consequently, the substance injected must have been absorbed by the glandular tissue, have passed by the circulation to the opposite side of the body, and exuded from the follicles of the opposite gland. As might be supposed, therefore, when the salt of iodine is injected directly into the veins, the interval of time is inappreciable between the performance of the injection and the appearance of the iodine in the parotid saliva.

The blood, accordingly, is constantly suffering important modifications in its passage through the circulation. A very curious calculation was recently made by Dr. Brown-Séquard, and published by him in the "Journal of Physiology" for April, 1858, regarding the quantity of fibrin produced and destroyed in the body day by day. The calculation is based upon a singular fact, established by the observations of Simon, Bernard, Lehmann, and Brown-Séquard, viz., that the venous blood coming from the kidneys and the liver is either destitute of fibrin, or contains this substance in much smaller proportion than arterial blood. The fibrin, therefore, disappears from the blood during its passage through these organs; and as it, nevertheless, continues to be found in the general mass of the blood in its ordinary proportion, viz., 2.5 parts per thousand, it is evident that a corresponding production of fibrin must take place somewhere in the body, by which its loss in the liver and kidneys is counterbalanced. The object of the calculation above referred to, is to determine the amount of fibrin thus daily produced and destroyed in the circulation.

Dr. Brown-Séquard assumes, in his estimate, that the left ventricle empties itself completely at each pulsation, and deduces the quantity of blood passing through the liver and kidneys from the ascertained calibre of the vessels supplying them. For reasons already given, however, it is perhaps better to depend upon the quantity of blood actually escaping, in a given time, from the divided vessels, as the basis of our calculation. We have found, in this way, that in a dog weighing 14½ pounds, the entire quantity of blood passing through the heart in a day is 247 pounds. The same estimate applied to a man of ordinary stature gives 2,380 pounds per day. The liver and kidneys respectively weigh, on the average, 29 and 4 parts per thousand of the entire weight. As these organs are, if anything, more vascular than most other parts of the body, it is safe to estimate the quantity of blood distributed to them as bearing at least the same proportion to the entire amount contained in the body. The whole quantity of blood, then, flowing through both the liver and kidneys per day is 78.5 pounds, or 550,000 grains. The daily quantity of fibrin, accordingly, which is destroyed in these organs and reproduced elsewhere, is 2.5 parts per thousand of the above, viz., 1,375 grains. When we remember that a pound of ordinary blood contains only 17.5 grains of fibrin, and that the whole amount of this substance contained in the body at any one time is certainly not over 355 grains, we find that the fibrin of the entire blood is destroyed and reproduced in the circulation at least three times over in the course of a single day.

However unexpected this result may be, it is nevertheless exactly parallel with certain other facts which have been familiar to us for many years. We know, for example, that 500 grains of urea are daily produced in the blood, and actually discharged with the urine; and yet the proportion of this substance, existing in the circulation at any one time, is so small that it can only be detected by operating on several pounds of blood at once, and is recognized by the microscopic measurement of its crystals. The entire quantity actually eliminated in twenty-four hours can, therefore, be accounted for only by the rapidity with which successive portions of blood pass through the kidneys, thus draining away the excrementitious matter as fast as it originates in other parts of the circulation.

Various other substances are produced and destroyed in this way, during the progress of the internal changes of waste and assimilation. The whole profession is familiar with the experiments, originated by Bernard, which show that sugar is constantly produced in the tissue of the liver, absorbed thence by the blood-vessels, carried away by the blood of the hepatic veins, and decomposed in the general current of the circulation. Lehmann has found, as the mean result of six observations on the dog, that the blood of the hepatic vein in this animal, contains sugar in the proportion of about eight parts per thousand, of the dry residue. This makes a little over 1.5 parts per thousand of the entire blood. Now, we know, from what has been said above, that the entire quantity of blood, passing through the liver per day, in the human subject, is not less than 69.02 pounds. The whole amount of sugar, therefore, produced in the liver, carried away from it by the hepatic blood, and destroyed in the circulation during twenty-four hours, is 792 grains, or a little over one ounce and a half.

These facts regarding the circulation of the blood, enable us to appreciate more readily the phenomena of another circulation constantly going on in the body, secondary in importance to that of the blood, but still much more active and abundant than we have hitherto supposed, viz., the lymphatic or absorbent circulation. Here, the movement of the fluids is not exactly a circulation, strictly speaking; since they move only in one direction, viz., from the circumference toward the centre. These fluids originate altogether by transudation and exosmosis from the substance of the tissues; and flow inward toward the heart, impelled mainly by this force of accumulation. From what we know of the rapidity with which these actions of endosmosis and exosmosis go on in the living body, we are ready to understand that the movement of the fluids thus produced by them may be very active; and yet the results of direct observation on this subject are again found to exceed considerably any of our previous estimates.

In May, 1857, a memoir was presented to the French Academy, giving an account of some experiments on the lymph and chyle of cows and oxen, performed by M. Colin, of the veterinary school at Alfort, and by M. Bérard, the distinguished professor of physiology of the Faculty of Medi cine at Paris, the news of whose death has been recently announced. These observers established a fistula of the thoracic duct, just as it emerges from the chest at the root of the neck, to join the great venous trunks in this situation. The experiments were performed mainly for the purpose of investigating another point in the physiology of the chyle, but gave also very remarkable results in regard to the quantity of the fluids passing through the thoracic duct, per day. These results I will give in the animated and descriptive language of M. Bérard himself.

"What I am going to say," he observes, "in answer to this objection, will produce a kind of stupefaction in the assembly. The chyle, this precious fluid, which some of those who hear me perhaps have never seen, and which physiologists can usually obtain only in little samples, so to speak,—how much of it do you imagine one of our oxen furnished, by his fistula, in twenty-four hours?—EIGHTY POUNDS" (*quarante litres*). "I confine myself," he adds, "within the limits of the truth."

M. Colin has also published these and other similar experiments in detail. From two experiments on the horse, extending over a period of twelve hours each, he calculates the quantity of fluid passing through the thoracic duct in this animal, as from twelve to fifteen thousand grains per hour, or between forty and fifty pounds per day. This quantity is still greater, according to his observation, in the ruminating animals. In a cow of medium size, the smallest quantity obtained, during an experiment of twelve hours' duration, was a little over 9,000 grains in fifteen minutes—that is, five pounds per hour, or 120 pounds per day. A little over 100 pounds was actually obtained in this manner, in another experiment, from a young bull with a fistula of the thoracic duct.

These estimates, I believe, are not exaggerated. By placing a canula in the thoracic duct of the dog, at the root of the neck, at various periods after feeding, I have obtained the following quantities of chyle: 3½ hours after feeding, 420 grains in an hour, in a dog weighing 24½ lbs.; 7 hours after feeding, 840 grains in an hour, in a dog weighing 54½ lbs.; 13 hours after feeding, 430 grains in two hours, in a dog weighing 31 lbs., and 18½ hours after feeding, 425 grains in an hour, in a dog weighing 32 pounds. The average of all these results gives the total quantity of lymph and chyle, during 24 hours, as very nearly 4½ per cent. of the entire weight of the animal; and this proportion, in a horse weighing 10 hundred weight, would be 45 pounds, substantially the same estimate as that obtained by Colin, in his experiments at Alfort.

By bringing together the quantities of the different animal fluids secreted and reabsorbed during 24 hours, as above ascertained, for a man weighing 140 lbs., we have the following table:—*

SECRETED AND REABSORBED DURING 24 HOURS.

| | | | |
|---|---|---|---|
| Saliva | 21,164 | grs. | 3.020 lbs. |
| Gastric juice | 98,000 | " | 14.000 " |
| Bile | 16,940 | " | 2.420 " |
| Pancreatic juice | 2,500 | " | 0.357 " |
| Lymph and chyle | 44,100 | " | 6.300 " |
| | | | 26.097 " |

* It will be observed that, in all these cases, the fluids are calculated at their *minimum* quantity, according to the present state of our knowledge.

From all these observations and their results, we gradually obtain a deeper insight into the nature and phenomena of the circulation as a whole. The capillary circulation, more especially, is one which has long presented to the mind of the physiologist many points very difficult of explanation. What is the nature of the force which keeps up this continuous movement of the blood? The only mechanical impulse, which can seriously influence it, is the force of the heart's contraction. But it is plain that the heart's impulse alone is not sufficient to explain the varied phenomena of the capillary circulation. It does not explain the congestion of a square inch of skin, from the action of a local cause, while the neighboring parts are unaffected, and the heart's action itself neither increased nor diminished. It does not explain the sudden excitement and vascularity of the digestive organs on the introduction of food, nor the periodical hemorrhage of the uterine mucous membrane in menstruation. Moreover, direct experiment shows conclusively that the heart's action is not the cause which produces the movement of the blood in the capillaries. If the portal vein and hepatic artery be snddenly obliterated by a ligature, the liver almost immediately grows pallid, and empties itself of blood in the course of a few seconds. Everything indicates that the capillary circulation is a local phenomenon, dependent on some force resident in the capillaries themselves, and in the neighboring tissues. From the facts which have now been brought together, I think we are better able to understand the exact nature of this force, and the mode in which it operates.

We find that the blood and the tissues are the seat of a constant and reciprocal process of endosmosis and exosmosis. The tissues absorb from the blood; the blood absorbs from the tissues. Consequently, the ingredients of the blood, and even its quantity, are changed while passing through the capillaries of an organ. The hepatic blood contains sugar, which did not exist in the blood of the portal vein. The blood which returns to the heart by the pulmonary veins, is less in quantity than that which passes out from it by the pulmonary artery; for the carbonic acid and watery vapor exhaled from the blood in the lungs weigh more than the oxygen which it absorbs in the same organs. For a similar reason, less blood returns from the kidneys by the renal veins than passes into them by the arteries, since the blood loses, during its passage, water, urea, and salts, which are discharged with the urine. On the other hand, the veins of the portal system bring away from the intestine a larger quantity of blood than is supplied by the mesenteric arteries; for the vessels absorb from the intestinal cavity the products of digestion, and thus become crowded with new ingredients. It is even maintained by a very careful and experienced observer,* that the entire quantity of blood undergoes incessant variations, corresponding with the different periods of digestion, and that, in the same animal, it is nearly twice as great during the period of active intestinal absorption as it is in the intervals of the digestive process.

The actual phenomena, therefore, of the capillary circulation may perhaps be best described in the following terms: *The blood which passes out of an organ by the veins is not the same blood which passed into it by the arteries.* The blood which arrives by the arteries is instantly seized and

---

* Bernard: *Liquides de l'Organisme.* Paris, 1859. Vol. i., p. 419.

acted upon by the tissues of the organ. Its fluid ingredients are taken up by absorption, and at the same time are replaced by others, exuded from the neighboring parts. It is not only certain ingredients of the circulating fluid, therefore, which are produced and destroyed in the interior of the body, but it would be a much more exact expression to say that the *entire blood* is constantly altered and reconstructed in its passage through different parts of the circulatory system. Every organ produces in it a different modification, and this modification is constantly repeated, as successive portions of blood arrive by the arteries and are carried away by the veins. The blood which passes off by the veins is accordingly to be regarded as supplied, in great measure, by the tissues of the organ itself—very much as a fluid secretion is exuded from the follicles of a gland, and carried off by the excretory duct—or as the lymph is absorbed from the substance of the tissues, and carried inward by the lymphatic vessels.

We find, therefore, that the digestive and other secretions, together with the lymph and chyle, give rise to a kind of internal circulation, or interchange of the animal fluids, secondary to the circulation of the blood; and that the whole amount of these fluids exuded and reabsorbed, in the human subject, is over twenty-five pounds per day. The incessant activity of the vital operations is accordingly one of the most singular phenomena of the living body, and it becomes still more remarkable when subjected to the test of direct examination and actual measurement.—*Tran. N. Y. Academy of Medicine.*

